# Epidemiology and risk factors of nasal carriage of *Staphylococcus aureus* CC398 in two distinct cohorts in France

**DOI:** 10.3389/fmicb.2022.1068420

**Published:** 2022-12-20

**Authors:** Kevin Bouiller, Abdeljalil Zeggay, Houssein Gbaguidi-Haore, Didier Hocquet, Catherine Chirouze, Xavier Bertrand

**Affiliations:** ^1^Service de Maladies Infectieuses et Tropicales – CHU Besancon, Besancon, France; ^2^UMR-CNRS 6249 Chrono-environnement, Université Bourgogne Franche-Comté, Besançon, France; ^3^Service d’Hygiène Hospitalière – CHU Besancon, Besancon, France

**Keywords:** human ST398, methicillin-susceptible *Staphylococcus aureus*, CC398, nasal carriage, *Staphylococcus aureus*

## Abstract

**Background:**

We aimed to determine the prevalence and factors associated with nasal carriage of *Staphylococcus aureus* CC398 in the community and among hospitalized patients.

**Methods:**

We conducted a prospective cohort study in a French university hospital and a cross-sectional study in the surrounding region.

**Results:**

From June 2019 to July 2020, 591 healthy blood donors (HBDs) and 647 hospitalized patients (HPs) were included. *S. aureus* CC398 was more prevalent in HBDs than in HPs (7.3% [5.3–9.7] vs. 3.8% [2.4–5.5], *p* = 0.006). Among *S. aureus* nasal carriers, the prevalence of CC398 isolates was 24.6% in HBDs and 18.3% in HPs (*p* = 0.19). No MRSA belonged to CC398. In multivariate analysis, prior antibiotic intake in the past year (OR 3.11 [1.37–7.06]) and active smoking (OR 3.01 [1.00–9.05]) were associated with *S. aureus* CC398 nasal carriage in the HBD cohort. A history of neurological disease was associated with nasal carriage (OR = 5.43 [1.21–24.2]), whereas an age between 82 and 90 years (OR 0.11 [0.02–0.54]) and diabetes (OR 0.18 [0.04–0.85]) were protective factors in the HP cohort. Contact with livestock was not a risk factor in either cohort.

**Conclusion:**

The prevalence of MSSA CC398 was higher in the community than hospitalized patients. Factors associated with nasal carriage of MSSA CC398 were primarily related to general preconditions. No environmental sources of exposure were identified.

## 1. Introduction

*Staphylococcus aureus* is a major cause of human morbidity and mortality worldwide, as well as a zoonotic pathogen (Price et al., 2012; Bai et al., 2022). *S. aureus* is a ubiquitous bacterium that is part of the human commensal flora. The mucosa of the anterior nasal cavity is the preferred site of colonization and has been characterized as the most common risk factor for subsequent *S. aureus* infection (Verhoeven et al., 2014). Nasal carriage depends on several factors: host-related factors (genetics, medical history, sex, etc.), environmental factors (such as seasonality), and factors specific to the bacteria (adhesion properties) (Verhoeven et al., 2014).

Clonal complex 398 (CC398) has acquired a special place within the species due to its emergence in the 2000s and its rapid spread in certain countries (Bouiller et al., 2020). *S. aureus* belonging to CC398 was first described in France among pig farmers and was subsequently found to cause a significant number of methicillin-resistant *S. aureus* (MRSA) infections in the Netherlands (and other parts of Europe), Asia, and North America (Stegger et al., 2011; Uhlemann et al., 2017). However, so-called livestock-associated *S. aureus* CC398 and human–associated *S. aureus* CC398 show a number of differences. The *SCCmec* cassette, tetracycline resistance (due to the *tetM* gene), and zinc-resistance genes are present mainly in livestock-associated *S. aureus* CC398, whereas most human-associated *S. aureus* is methicillin susceptible and solely shows resistance to macrolides (encoded by the *ermT* gene) (Uhlemann et al., 2012; Chroboczek et al., 2013). Another difference is the presence of an immune evasion cluster (IEC), supported by the prophage of integrase group 3 (φSa3), in isolates from humans. The acquisition of the φSa3 prophage is specific to the human lineage of *S. aureus* CC398 (Uhlemann et al., 2012; van der Mee-Marquet et al., 2013).

Since 2010, several studies have described an increasing prevalence of methicillin-sensitive *S. aureus* (MSSA) CC398 in bloodstream infections (Sauget et al., 2019; Gu et al., 2020; Mama et al., 2021). CC398 isolates may be associated with a higher risk of mortality than those of other clonal complexes, especially for patients with comorbidities or neurological diseases (Bouiller et al., 2016). The proportion of MSSA CC398 among MSSA responsible for bacteremia in our hospital increased four-fold to 15% between 2009 and 2014 (Sauget et al., 2019). However, the location of acquisition (community or hospital) of these strains is still unclear, due to the lack of screening data upon admission. Unlike the acquisition of MRSA CC398 related to contact with livestock, the risk factors for nasal carriage of MSSA CC398 have not been well studied. A study in China showed nasal carriage of MSSA CC398 to be associated with the presence of household members practicing a healthcare profession, suggesting nosocomial origin (Yan et al., 2015).

The aims of the study were to determine the prevalence of nasal carriage of *S. aureus* CC398 in the community and in hospitalized patients and to evaluate the factors associated with carriage.

## 2. Materials and methods

### 2.1. Setting and study period

We conducted a prospective cohort study from June 2019 to July 2020 in the Besançon University Hospital and a cross-sectional study in the surrounding region (Franche-Comte, East of France). The institution has 1,200 beds, with approximately 50,000 admissions and 320,000 patient days annually. The Franche-Comte region has approximately 1 million inhabitants, with a population density of 73 inhabitants/km^2^. The density of pigs is relatively low, with the highest density around 10 pigs/km^2^ (see Supplementary material).

### 2.2. Community cohort

**Figure 1:**
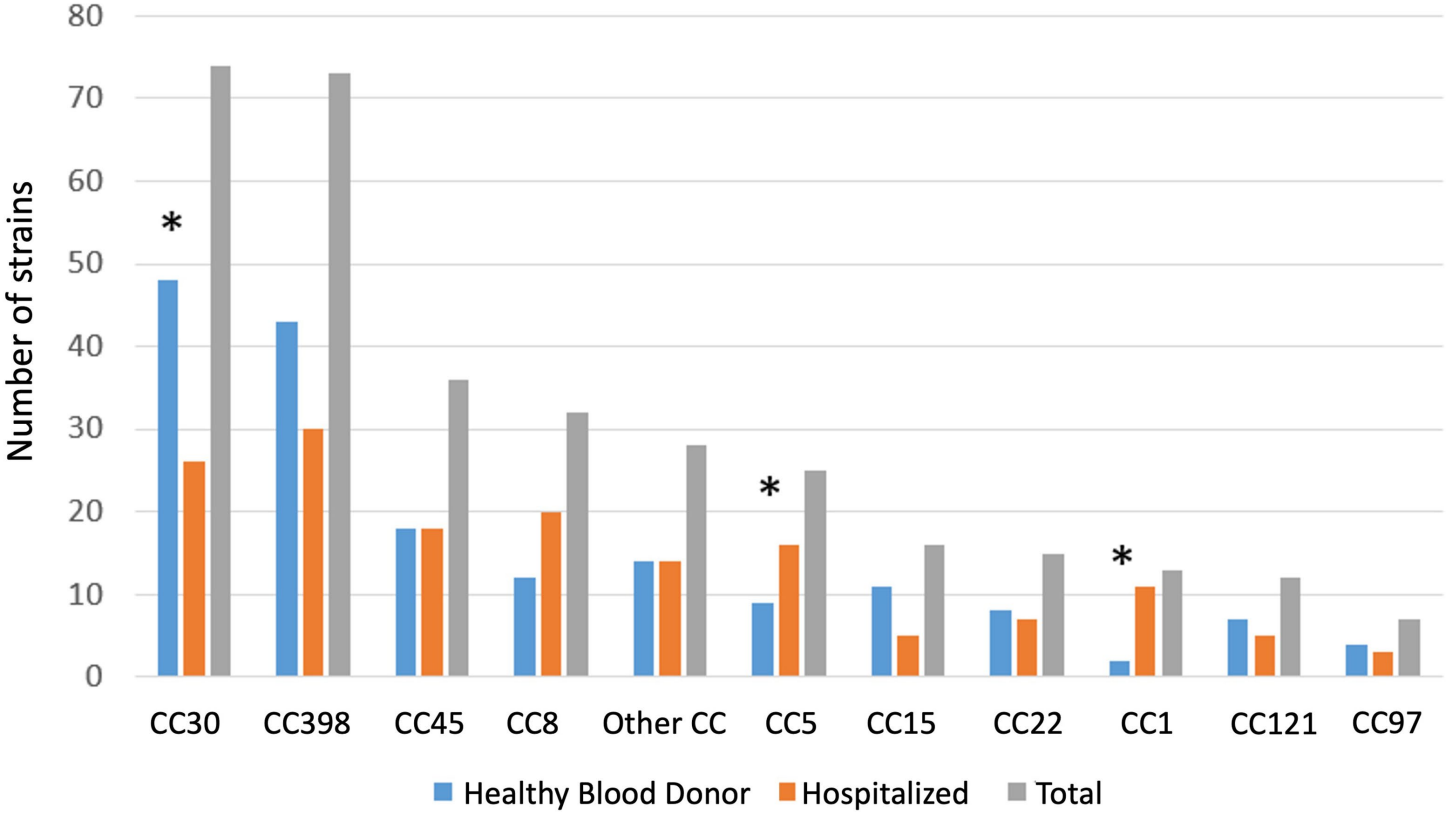
Distribution of *Staphylococcus aureus* clonal complex in hospitalized patients and healthy blood donor. Other: CC7 (*n* = 5), CC182 (*n* = 3), CC1640 (*n* = 2), CC59 (*n* = 3), CC20 (*n* = 2), unknown (*n* = 3), CC10, CC12, CC80, CC88, CC101, CC152, CC395, CC707, CC3803, CC106, (one each). * *p* < 0.05.

*Staphylococcus aureus* nasal carriage in the community was assessed by performing a nasal swab on voluntary adult blood donors over 1 week every 3 months in different cities of the Franche-Comte region (Supplementary Data; Figure 1). Demographic data, the medical history, and data pertaining to factors potentially related to *S. aureus* nasal carriage were prospectively collected.

### 2.3. Hospitalized patients

*S. aureus* nasal carriage in hospitalized patients was assessed by performing a nasal swab on adult patients admitted to the geriatrics, infectious diseases, hematology, and dermatology departments at the University hospital of Besancon. A sample was collected upon admission and on day 7 of hospitalization or upon discharge from the hospital (if before day 7). In addition to the information collected on patients in the community, the McCabe score and information concerning antibiotic intake during hospitalization were collected.

### 2.4. Detection of *Staphylococcus aureus* clonal complex 398

All samples were cultured on Columbia agar with 5% horse blood (Thermo Fischer Scientific, Breda, Netherlands). Identification of *S. aureus* was carried out by matrix-assisted laser desorption/ionization time-of-flight mass spectrometry (MALDI-TOF MS) Microflex LT (Bruker Daltonik GmbH, Bremen, Germany), with a log score value ≥2 according to the manufacturer’s recommendations. Susceptibility to 16 antimicrobial agents (cefoxitin, tetracycline, kanamycin, tobramycin, gentamicin, erythromycin, clindamycin, pristinamycin, norfloxacin, ofloxacin, cotrimoxazole, fosfomycin, fusidic acid, nitrofurantoin, linezolid, and rifampicin) was determined using the disk diffusion method according to 2020 European Committee on Antimicrobial Susceptibility Testing recommendations (The European Committee on Antimicrobial Susceptibility Testing, 2020). Breakpoint tables for interpretation of MICs and zone diameters, version 10.0, 2020). Inducible resistance of clindamycin was not performed.

### 2.5. Whole-genome sequencing

*S. aureus* isolates were sequenced using an Illumina NextSeq instrument, which generates 150-bp paired-end reads with a mean coverage of 80X. Bioinformatics analysis was performed using Galaxy. *De novo* assembly of the genomes of all isolates was performed using SPAdes v.3.11.1.

### 2.6. Molecular typing methods

Multi-locus Sequence Typing of *S. aureus* isolates was determined *in silico* using the Enright scheme and STs were grouped into CCs (Enright et al., 2000). *S. aureus* CC398 strains were clustered using wgMLST scheme.

### 2.7. Ethics statement

The protocol was approved by the Research Board of CPP Sud-Ouest et Outre-Mer 1 (CPP 1–18-92 / SI 1780). Informed consent was obtained from each participant at the time of nasal screening.

### 2.8. Statistical analysis

The expected prevalence (Pr) of *S. aureus* CC398 in the community and hospital settings has been estimated to be 2% (20% *S. aureus* nasal carriage in France (den Heijer et al., 2013) and 10% *S. aureus* CC398 among hospitalized patients in Besancon (Bouiller et al., 2016)). An alpha risk of 5% and inclusion of 1,000 blood donors and 1,000 hospitalized patients yields an absolute precision for prevalence of approximately 0.87% (Pr = 2% ± 0.87) for each of the two groups.

Continuous variables are presented as medians with interquartile ranges (IQRs) and categorical data as percentages. Comparisons of continuous variables were performed using the Student t-test or the Mann–Whitney U test. Categorical variables were compared using χ^2^ or Fischer exact tests, as appropriate. Factors independently associated with the prevalence of *S. aureus* CC398 nasal carriage were determined using logistic regression models. Multivariable models were built using a backward elimination process with independent variables with a *value of p* <0.10 in univariable analysis. All analyses were performed using the software package Stata, version 14.1 (Stata Corp., College Station, TX, United States). A *value of p* <0.05 was considered statistically significant.

## 3. Results

### 3.1. Inclusion of patients

From June 2019 to July 2020, 591 healthy blood donors (HBDs) and 647 hospitalized patients (HPs) were included. Sample collection in the hospital was interrupted for 3 months due to the Covid-19 pandemic (between March and June 2020). All HPs were screened upon admission but only 294 (45.4%) were screened on day 7 or upon discharge.

### 3.2. Prevalence of *Staphylococcus aureus* and *Staphylococcus aureus* CC398

Among nasal swab samples from HBDs, 175 (29.6%) were positive for *S. aureus*. Only 2 HBDs were positive for MRSA (1.1%). CC30 was the most common CC (*n* = 48, 27%), followed by CC398 (*n* = 40, 24%), CC45 (*n* = 18, 10%), and CC8 (*n* = 12, 7%) (Figure 1).

Among HPs, 138 (21.3%) nasal swabs were positive for *S. aureus* upon admission, of which seven were MRSA (5%). Among 295 HPs with a second swab, 58 showed nasal colonization with *S. aureus*. Among 238/295 HPs with an initial negative sample, 18 showed *S. aureus* colonization on day 7. Whole genome sequencing data were not available for 8 HPs due to sequencing problems (seven patients at inclusion and one patient on day 7). Overall, the genomes of 148 *S. aureus* isolates from HPs were analyzed. CC398 was the most frequent CC (*n* = 30/148, 20.3%), followed by CC30 (*n* = 26, 17.6%), and CC8 (*n* = 2 0, 13.5%) (Table 1).

**Table 1:** Prevalence of nasal carriage of *S. aureus* and *S. aureus* CC398 in healthy blood donors and hospitalized patients.

	Community (Healthy Blood donor)	Hospitalized			*p* (C *vs* H admission)	*p* (C *vs* H day 7)
	At inclusion, *N* = 591	At entry, *N* = 647	At day 7, *N* = 295	Acquisition**, *N* = 237		
*S. aureus*, *n*	175	138	58	18		
Prevalence, % (IC95%)	29.6 (26–33.5)	21.3 (18.2–24.7)	19.4 (15–24.4)	7.6 (4.5–11.7)	0.001	0.001
MRSA, n	2	7	7	3		
Prevalence, % (IC95%)	0.3 (0.04–1.2)	1.1 (0.4–2.2)	2.4 (0.1–4.8)	1.3 (0.3–3.6)	0.18	0.008
Prevalence MRSA/SA, % (IC95%)	1.1 (0.1–4.1)	5 (2.1–10.2)	12.3 (5.1–23.7)	16.7 (3.6–44.4)	0.047	0.001
MSSA, n	173	131	50	15		
Prevalence, % (IC95%)	29.3 (25.6–33.1)	20.2 (17.2–23.6)	17.3 (12.9–21.8)	6.3 (3.6–10.2)	<0.001	<0.001
Prévalence SA CC398, n	43/591	24/640*	16/293*	6/237		
%, (IC95%)	7.3 (5.3–9.7)	3.8 (2.4–5.5)	5.5 (3.2–8.7)	2.5 (0.9–5.4)	0.006	0.31
Prevalence SA CC398 /SA	43/175	24/131	16/56	6/18		
%, (IC95%)	24.6 (18.4–31.6)	18.3 (12.1–26)	28.6 (17.3–42.2)	33.3 (13.3–59)	0.19	0.55
Prevalence SA CC398 /MRSA	0	0	0	0	–	–
Prevalence SA CC398/MSSA	43/173	24/125	16/50	6/15		
%, (IC95%)	24.9 (18.6–32)	19.2 (12.7–27.2)	32 (19.5–46.7)	40 (16.3–67.7)	0.25	0.31

SA*, Staphylococcus aureus*; CC, Clonal complex; C, community; H, hospitalized; MSSA, Methicillin susceptible *Staphylococcus aureus*; MRSA, Methicillin resistant *Staphylococcus aureus.*

* Genome sequencing of 8 SA (7 at inclusion and 1 at day 7) strains were not available.

** Nasal colonization negative at inclusion, but positive at day 7.

The prevalence of *S. aureus* was significantly higher among HBDs than HPs (29.6% [95% CI, 26–33.5] vs. 21.3% [18.4–24.9], *p* = 0.001). Similarly, *S. aureus* CC398 was more prevalent among HBDs than HPs (7.3% [5.3–9.7] vs. 3.8% [2.4–5.5], *p* = 0.006). CC 30 was also more common among HBDs than HPs (8.1% vs. 4%, *p* < 0.022), but CC1 and CC5 were less frequently found (0.3% vs. 1.7%, *p* < 0.001, and 1.3% vs. 2.5%, *p* = 0.04, respectively) (Table 1).

### 3.3. Factors independently associated with nasal carriage of *Staphylococcus aureus* CC398 among healthy blood donors

Among HBDs, *S. aureus* CC398 carriers (*n* = 43) were younger (29 vs. 44 years, *p* = 0.02), more often had prior antibiotic intake in the past year (42% vs. 20%, p = 0.001), chronic dermatological disease (23% vs. 8.1%, p = 0.001), and estroprogestative contraception (28.6% vs. 13.6%, *p* = 0.009) than non-carriers of CC398. In multivariate analysis, being younger than 24 years of age (OR 2.00 (1.02–3.94)), prior antibiotic intake in the past year (OR = 2.46 (1.29–4.72)), and chronic dermatological disease (OR 2.28 (1.01–5.12)) were associated with *S. aureus* CC398 nasal carriage (Table 2).

**Table 2:** Comparison of patients with nasal carriage of *S. aureus* CC398 with patients with nasal carriage of other clonal complex or no carriage of *S. aureus.*

Characteristics		Healthy Blood Donors (HBD) N=591			Hospitalised N=640			
		Non *S. aureus* CC398*, n=548	*S. aureus* CC398, n=43	*p*	Non *S. aureus* CC398*, n=616	*S. aureus* CC398, n=24	*p*	p**
**Demographic**								
Age, med [Q25-75], year		44.3 (25.5-57.8)	28.9 (19.6-53.2)	0.02	82.5 (70-89.1)	80.7 (56.5-92.4)	0.99	<0.001
HBD	Hospitalised							
<24 y	<69 y	127/536 (23.7)	17/42 (40.5)	0.02	147/614 (23.9)	8/24 (33.3)	0.29	
[24-43[	[69-82[	134/536 (25)	10/42 (23.8)	0.86	140/614 (22.8)	5/24 (20.8)	0.82	
[43-57[	[82-90[	132/536 (24.6)	8/42 (19.1)	0.42	194/614 (31.6)	2/24 (8.3)	0.01	
≥57 y	≥90 y	143/536 (26.7)	7/42 (16.7)	0.15	133/614 (21.7)	9/24 (37.5)	0.07	
Female sex		240/546 (44)	22/43 (51.2)	0.36	337/614 (54.9)	16/24 (66.7)	0.26	0.22
Body mass index, kg/m^2^		24.3 (22.1-27)	24.2 (22.1-27.2)	0.73	24.6 (21-28.7)	23.1 (20.7-27)	0.40	0.43
**Occupation**								
HCW		46/535 (8.6)	6/42 (14.3)	0.22	8/540 (1.5)	0/23	>0.99	0.08
Agriculture		35/535 (6.5)	3/42 (7.1)	0.75	14/540 (2.6)	0/23	>0.99	0.55
Other		454/535 (84.9)	33/42 (78.6)	0.28	518/540 (95.9)	23/23 (100)	>0.99	0.02
**Habitus**								
Active smoking		63/497 (12.7)	8/37 (21.6)	0.12	47/346 (13.6)	1/12 (8.3)	>0.99	0.42
Alcohol		22/492 (4.5)	2/37 (5.4)	0.68	31/290 (10.7)	0/10	0.61	>0.99
Vegeterian		12/541 (2.2)	1/43 (2.3)	>0.99	6/289 (2.1)	0/10	>0.99	>0.99
Animals at home		315/546 (57.7)	22/43 (51.2)	0.41	94/291 (32.3)	4/10 (40)	0.73	0.73
Cat, dog		297/546 (54.4)	21/43 (48.8)	0.48	87/291 (29.9)	3/10 (30)	>0.99	0.32
Rabbit		32/545 (5.9)	2/43 (4.70)	>0.99	7/290 (2.4)	2/10 (20)	0.03	0.16
Weasel		2/545 (0.4)	0/43	>0.99	3/290 (1)	0/10	>0.99	-
Swine		0/545	0/43	-	1/290 (0.3)	0/10	>0.99	-
Other		48/545 (8.8)	1/43 (2.3)	0.14	19/287 (6.6)	0/10	>0.99	>0.99
Outdoor activities		316/541 (58.4)	25/43 (58.1)	0.97	70/285 (24.6)	2/10 (20)	>0.99	0.04
Recent trip abroad (< 1 year)		131/546 (24)	10/43 (23.3)	0.91	41/293 (14)	3/10 (30)	0.17	0.69
Number of persons living in the same household								
- ≤1		128/478 (26.8)	8/36 (22.2)	0.55	317/415 (76.4)	14/17 (82.4)	0.77	0.02
- [2-3]		230/478 (48.1)	20/36 (55.6)	0.39	77/415 (18.6)	1/17 (5.9)	0.33	0.03
- >3		120/478 (25.1)	6/33 (18.2)	0.70	21/415 (5.1)	2/17 (11.8)	0.23	>0.99
HCW living in the same household		30/493 (6.1)	3/39 (7.7)	0.73	47/393 (12)	3/16 (18.8)	0.43	>0.99
Person living in the same household and hospitalized (<1 year)		51/532 (9.6)	5/41 (12.2)	0.59	75/396 (18.9)	4/16 (25)	0.52	0.60
Antibiotic intake (<1 year)		107/533 (20.1)	17/41 (41.5)	0.001	258/399 (64.7)	9/13 (69.2)	>0.99	0.32
Recent hospitalisation (< 1 y)		22/533 (4.1)	1/41 (2.4)	>0.99	297/602 (49.3)	9/24 (37.5)	0.26	0.003
**Comorbidities**								
Diabete		1/534 (0.2)	0/43	>0.99	134/597 (22.5)	2/23 (8.7)	0.20	0.12
Malignancy		1/377 (0.3)	0/34	>0.99	158/600 (26.3)	4/23 (17.4)	0.47	0.02
Hypertension		20/538 (3.7)	2/42 (4.8)	0.67	335/599 (55.9)	8/23 (34.8)	0.045	0.003
Chronic liver disease		0/376	0/32	-	9/595 (1.5)	0/22	>0.99	-
Chronic heart disease		0/391	0/35	-	114/598 (19.1)	6/23 (26.1)	0.40	0.002
Peripheral arteriopathy		1/380 (0.3)	0/35	>0.99	132/594 (22.2)	7/23 (30.4)	0.36	0.001
Chronic pulmonary disease		0/387	0/35	-	70/600 (11.7)	1/23 (4.4)	0.50	0.40
Chronic renal insufficiency		0/392	0/35	-	70/597 (11.7)	3/23 (13)	0.75	0.06
Neurological disease		1/386 (0.3)	0/35	>0.99	120/593 (20.2)	5/22 (22.7)	0.78	0.006
Chronic dermatologic disease		44/541 (8.1)	10/43 (23.3)	0.001	60/287 (20.9)	4/10 (40)	0.23	0.43
HIV		0/367	0/34	-	4/598 (0.4)	0/23	>0.99	-
Intravenous drug use		0/379	0/35	-	3/217 (1.4)	0/5	>0.99	-
Allergy		104/531 (19.6)	13/42 (31)	0.08	80/329 (24.3)	3/12 (25)	>0.99	>0.99
Hormonal contraceptive		68/500 (13.6)	12/42 (28.6)	0.009	1/558 (0.2)	0/22	>0.99	0.005
Ultimately fatal disease (McCabe score)		0	0	-	51/560 (9.1)	2/23 (8.7)	0.78	
**Hospitalisation management**								
Antibiotic therapy during hospitalisation		-	-		314/599 (52.4)	9/23 (39.1)	0.21	
In hospital mortality		-	-		47/616 (7.6)	1/24 (4.2)	>0.99	

** Comparison characteristics of hospitalized patients with nasal carriage of *S.aureus* CC398 vs healthy blood donors with nasal carriage of *S.aureus* CC398.

Among HBDs with *S. aureus* nasal carriage (*n* = 175), factors associated with nasal carriage of *S. aureus* CC398 were the prior intake of antibiotic therapy in the past year (OR 3.11 [1.37–7.06] and active smoking (3.01 [1.00–9.05]) (Table 3).

**Table 3:** Comparison of patients with nasal carriage of *S. aureus* CC398 with patients with nasal carriage of other clonal complex.

**Characteristics**		**Healthy Blood Donors (HBD)** **N=591**			**Hospitalised, N=640**		
		**SA other CC, n=132**	**SA CC398,** **n=43**	***p***	**SA other CC,** **n=107**	**SA CC398,** **n=24**	***p***
**Demographic**							
Age, med [Q25-75], year		44.3 (25.5-57.8)	28.9 (19.6-53.2)	0.02	82.5 (70-89.1)	80.7 (56.5-92.4)	0.99
HBD	Hospitalised						
<24 y	<69 y	42/127 (33.1)	17/42 (40.5)	0.38	25/107 (23.4)	8/24 (33.3)	0.31
[24-43[	[69-82[	38/127 (29.9)	10/42 (23.8)	0.45	18/107 (16.8)	5/24 (20.8)	0.64
[43-57[	[82-90[	25/127 (19.7)	8/42 (19.1)	0.93	42/107 (39.3)	2/24 (8.3)	0.004
≥57 y	≥90 y	22/127 (17.3)	7/42 (16.7)	0.92	22/107 (20.6)	9/24 (37.5)	0.08
Female sex		49/131 (37.4)	22/43 (51.2)	0.11	56/106 (52.8)	16/24 (66.7)	0.22
Body mass index, kg/m^2^		24.7 (21.9-28)	24.2 (22.1-27.2)	0.46	24.6 (19.8-29)	23.1 (20.7-27)	0.62
**Occupation**							
HCW		10/129 (7.8)	6/42 (14.3)	0.21	0/92	0/23	-
Agriculture		14/129 (10.9)	3/42 (7.1)	0.77	2/92 (2.2)	0/23	>0.99
Other		105/129 (81.4)	33/42 (78.6)	0.69	90/92 (97.8)	23/23 (100)	>0.99
**Habitus**							
Active smoking		10/123 (8.1)	8/37 (21.6)	0.02	6/60 (10)	1/12 (8.3)	>0.99
Alcohol		3/122 (2.5)	2/37 (5.4)	0.33	8/53 (15)	0/10	0.34
Vegeterian		3/129 (2.3)	1/43 (2.3)	>0.99	2/52 (3.9)	0/10	>0.99
Animals at home		86/132 (65.2)	22/43 (51.2)	0.10	13/52 (25)	4/10 (40)	0.33
Cat, dog		81/132 (61.4)	21/43 (48.8)	0.15	12/52 (23.1)	3/10 (30)	0.69
Rabbit		8/132 (6.1)	2/43 (4.7)	>0.99	1/53 (1.9)	2/10 (20)	0.07
Weasel		0/132	0/43	-	0/52	0/10	-
Swine		0/132	0/43	-	0/52	0/10	-
Other		14/132 (10.6)	1/43 (2.3)	0.12	2/51 (3.9)	0/10	>0.99
Outdoor activities		70/132 (53)	25/43 (58.1)	0.56	9/49 (18.8)	2/10 (20)	>0.99
Recent trip abroad (< 1 year)		27/132 (20.5)	10/43 (23.3)	0.70	3/50 (6)	3/10 (30)	0.05
Number of persons living in the same household							
- ≤1		29/118 (24.6)	8/36 (22.2)	0.77	55/71 (77.5)	14/17 (82.4)	>0.99
- [2-3]		51/118 (43.2)	20/36 (55.6)	0.19	10/71 (14.1)	1/17 (5.9)	0.68
- >3		38/118 (32.2)	8/36 (22.2)	0.25	6/71 (8.5)	2/17 (11.8)	0.65
HCW living in the same household		9/117 (7.7)	3/39 (7.7)	>0.99	9/70 (12.9)	3/16 (18.8)	0.69
Person living in the same household and hospitalized (<1 year)		13/129 (10.1)	5/41 (12.2)	0.70	14/71 (19.7)	4/16 (25)	0.73
Antibiotic intake (<1 year)		25/128 (19.5)	17/41 (41.5)	0.005	48/67 (70.6)	9/13 (69.2)	>0.99
Recent hospitalisation (< 1 y)		3/127 (2.4)	1/41 (2.4)	>0.99	61/106 (57.6)	9/24 (37.5)	0.08
**Comorbidities**							
Diabete		0/128	0/43	-	29/103 (28.2)	2/23 (8.7)	0.06
Malignancy		0/99	0/34	-	27/104 (26)	4/23 (17.4)	0.59
Hypertension		4/128 (3.1)	2/42 (4.8)	0.64	55/103 (53.4)	8/23 (34.8)	0.11
Chronic liver disease		0/99	0/32	-	3/104 (2.9)	0/22	>0.99
Chronic heart disease		0/103	0/35	-	23/104 (22.1)	6/23 (26.1)	0.68
Peripheral arteriopathy		0/100	0/35	-	24/103 (23.3)	7/23 (30.4)	0.47
Chronic pulmonary disease		0	0	-	16/104 (15.4)	1/23 (4.4)	0.31
Chronic renal insufficiency		1/103 (1)	0/35	-	20/104 (19.2)	3/23 (13)	0.77
Neurological disease		1/103 (1)	0/35	>0.99	8/102 (7.8)	5/22 (22.7)	0.04
Chronic dermatologic disease		16/129 (12.4)	10/43 (23.3)	0.09	16/56 (28.6)	4/10 (40)	0.47
HIV		0/98	0/34	-	1/103 (1)	0/23	>0.99
Intravenous drug use		0/97	0/35	-	0/39	0/5	-
Allergy		34/128 (26.6)	13/42 (31)	0.58	10/57 (17.5)	3/12 (25)	0.69
Hormonal contraceptive		19/118 (16.1)	12/42 (28.6)	0.08	0/95	0/22	-
Ultimately fatal disease (McCabe score)		0	0	-	7/99 (7.1)	2/23 (8.7)	0.57
**Hospitalisation management**							
Antibiotic therapy during hospitalisation		-	-		51/103 (49.5)	9/23 (39.1)	0.37
In hospital mortality		-	-		9/107 (8.4)	1/24 (4.2)	0.69

### 3.4. Factors independently associated with nasal carriage of *Staphylococcus aureus* CC398 among hospitalized patients at admission

Patients with *S. aureus* CC398 carriage (*n* = 24) were less often between 82 and 90 years of age (8.3% vs. 31.6%, *p* = 0.01) and less often had hypertension (34.8% vs. 55.9%, *p* = 0.045) than non-carriers of CC398. In multivariate analysis, no factors were associated with *S. aureus* CC398 nasal carriage.

Among all patients with *S. aureus* nasal carriage (*n* = 131), a history of neurological disease was associated with *S. aureus* CC398 nasal carriage (OR 5.43 [1.21–24.2], *p* = 0.03), whereas an age between 82 and 90 years (OR 0.11 [0.02–0.54]) and diabetes (OR 0.18 [0.04–0.85]) were protective factors against nasal carriage of *S. aureus* CC398.

### 3.5. Antibiotic resistance and prophage φSa3 of *Staphylococcus aureus* CC398

Concerning antibiotic resistance, among all samples (175 HBDs, 138 HPs at inclusion and 18 HPs at day 7), we found 12/331 (3.6%) MRSA isolates. Among HPs, the prevalence of MRSA upon admission was 7/138 (5.1%) versus 2/591 (1.1%) among the HBDs (*p* = 0.047). Among HPs with a second nasal swab, 3/238 (1.67%) were not colonized at first inclusion, but the second sample was positive for MRSA.

*S. aureus* CC398 isolates were only resistant to erythromycin in 84% of cases but susceptible to all other antibiotics, in particular, methicillin and tetracycline. The *erm(T)* gene was correlated to the phenotypic erythromycin resistance. None other macrolides resistance genes were present. Resistance to erythromycin was significantly associated with CC398 relative to other CCs (84% vs. 13%, *p* < 0.001). All *S. aureus* CC398 isolates carried the φSa3 prophage, and none carried the *tet(M)* and *Crzc* zinc-resistance genes (see Supplementary data).

## 4. Discussion

### 4. 1. Summary of the principal findings

We have performed the first study to evaluate the prevalence of nasal carriage of *S. aureus* CC398 in two cohorts: healthy blood donors (considered as healthy community patients) and hospitalized patients, in France. We found a higher nasal carriage of *S. aureus*, in particular, *S. aureus* CC398, for HBDs than HPs. However, the proportion of nasal carriage of CC398 among HPs on day 7 was similar to that among HBDs. No MRSA isolates were included in CC398. Factors associated with nasal carriage of CC398 differed between the two cohorts. Antibiotic use in the previous year and active smoking were associated with nasal carriage of CC398 in the community, whereas neurological disease, the absence of diabetes, and an age other than between 82 and 90 years were associated with nasal carriage among HPs. These findings suggest that the high rate of colonization was related to the local epidemiology of *S. aureus* CC398, as well as to host-associated factors. Another important finding was the absence of MRSA CC398 in our cohorts.

### 4.2. Distribution of *Staphylococcus aureus* clonal complexes

The population structure of *S. aureus* carriage isolates in the two cohorts revealed the dominance of two CCs, CC398 and CC30, representing 52% of all CCs in the HBD cohort and 37% in the HP cohort. While CC30 and CC398 were more frequently found among HBDs than HPs, CC5 and CC1 were more frequently found among HPs than HBDs. The observed diversity of CCs is consistent with previous findings among clinical MSSA strains (Valour et al., 2014; Yan et al., 2015; Bonnet et al., 2018). Similarly, *S.aureus* CC8, CC7 and CC5 were more prevalent in a cohort of German hemodialysis patients compared to healthy patients (22% vs. 10, 14% vs. 5, 6% vs. 3% respectively), whereas CC30 was underrepresented (11% vs. 21%) (Scheuch et al., 2019). Ultimately, the different clonal distribution is in agreement with the general observation that *S. aureus* colonization characteristics differ in the general population and in patients that are in frequent contact with health care system (hospital or nursing homes for the elderly). Moreover, *S. aureus* lineage has different specific set of virulence factors, such as adhesins, toxins and immune evasion cluster which could influence the diversity of *S. aureus* colonization in different population.

### 4.3. Prevalence of nasal carriage of *Staphylococcus aureus* CC398

Our results show the predominant position of CC398 in *S. aureus* nasal carriage both in the hospital setting and in the community, previously reported in blood stream infections from 2010 to 2017 in our hospital, with a prevalence among *S. aureus* strains reaching 20% in 2017 (Sauget et al., 2019). The high prevalence of this clone has also been reported in other institutions in Europe and throughout the world (Bouiller et al., 2020; Mama et al., 2021). However, such a high prevalence has been mostly reported in France and China (Bouiller et al., 2020).

In France, the geographical distribution of *S. aureus* CC398 was shown to be heterogeneous, depending on the region. In a study on MSSA bone and joint infection, the prevalence of CC398 varied from 3 to 23.5% (Valour et al., 2014).

A recent systematic review of the prevalence of nasal carriage of *S. aureus* found very low rates of MSSA CC398 among healthy people (< 1%) (with *spa* type t571, human-related clade) and farmers (1.8%) (t034 and t011, livestock-related clade) (Abdullahi et al., 2021). Only two French studies were included, none reported the distribution of MSSA CCs, and 0/16 MRSA strains were related to CC398 (den Heijer et al., 2013).

The results of other European studies have been variable, depending on the country, year of the study, and characteristics of the included patients (healthy patients, hospitalized patients, farmers). In Portugal, the rate of nasal carriage of MSSA CC398 among nursing students and the homeless population was 11 and 30% among the *S. aureus*-positive population between November 2016 and January 2018, respectively (Conceição et al., 2017). In Germany, in a study performed in 2011, *spa* type t011 (CC 398) was the predominant clone in MRSA nasal carriage (41%) among healthy people (ambulatory department). However, only 1% of MSSA strains belonged to CC398 (*spa* type t011, t034, t1451, t3423) (Becker et al., 2017).

A cross sectional study in two cities in China was performed with nasal swabs of 2,448 healthy volunteers. *S. aureus* MLVA complex 398 was the most prevalent complex, representing 21% of all isolates (Yan et al., 2015). For hospitalized patients, CC398 was the second most common CC among all isolates of *S. aureus* in seven nursing homes in Shanghai (20%), after CC1 (29%). It represented 31% of MRSA isolates and 17% of MSSA isolates (Zhang et al., 2015).

The absence of MRSA belonging to CC398 in our study can be explained by the low number of farmers in the two cohorts (6.6% among HBDs and 2.5% among HPs) and the absence of an human-adapted MRSA CC398 clone. A relationship between pig density and MRSA CC398 has been reported, with the highest area had 247 pigs/km^2^ in a study in Spain and 4,700 pigs/km^2^ in a study in Netherlands (van Loo et al., 2007; Ceballos et al., 2019). In Besancon area, the highest pig density is around 10 pigs/km^2^ in 2017, which could explain the absence of MRSA CC398 in our study.

### 4.4. Factors associated with *Staphylococcus aureus* CC398

Exposure to animals, in particular livestock, is a known risk factor for MRSA colonization. MRSA CC398 has been primarily reported for patients exposed to swine. This clade is often resistant to tetracycline. On an other hand, human clades are MSSA and erythromycin resistant in patients without contact with animals (Bouiller et al., 2020, 398). The absence of contact with swine in our study and the high rate of erythromycin resistance suggests that the *S. aureus* CC398 isolates are part of the human clade of CC398. Interestingly, the use of antibiotics in the previous year was associated with *S. aureus* CC398 nasal carriage. Previous studies have reported that antibiotic intake can increase the proportion of MRSA but not MSSA carriage (Forster et al., 2013; Mehraj et al., 2016). One explanation could be the high erythromycin-resistance rate of MSSA CC398 strains, which was as high as that of MRSA strains. Indeed, risk factors reported for community acquired MRSA colonization include the previous use of quinolones or macrolides (Cookson et al., 2011). Unfortunately, we did not record the type of antibiotic used in the previous year.

The association between smoking and *S. aureus* nasal carriage is a subject of debate. A number of authors have suggested that cigarette smoke has bactericidal activity and increases immune activity associated with smoking-induced hypoxia, whereas others have shown that smokers are more frequently colonized than non-smokers and cessation from smoking improves the clearance of *S. aureus* in an experimental model (Olsen et al., 2012; Cole et al., 2018). However, the relationship between smoking and nasal carriage with different *S. aureus* CCs has not been studied.

In China, factors associated with MVLA complex 398 strains were shown to be the place of residence (Harbin city) (OR = 1.77) and household members practicing a profession in healthcare (OR = 3.69). However, the origin of the clone (livestock clade or human clade) was not reported. The methicillin resistance of one strain could suggest an animal origin (Yan et al., 2015). In our study, no specific location was associated with *S. aureus* CC398 colonization. Franche-Comté is a rural region with a large geographical area. The lack of a specific location can be explained by the interconnection of cities in the same region.

We show various risk factors to be associated with *S. aureus* CC398 in HPs. Although being older is a well-known protective factor, diabetes has been reported as a risk factor for *S. aureus* nasal carriage. This finding was possibly associated with the polymorphism in the vitamin D receptor gene in patients with diabetes (Panierakis et al., 2009). However, this association has been reported more specifically for MRSA than MSSA (Forster et al., 2013). Moreover, in a study of patients with diabetic foot-ulcer and nasal swabs, *S. aureus* CC398 was reported in 9.9% (*n* = 10) of nares but 20% (*n* = 13) of diabetic foot ulcers. Although it was the third most frequent CC in nares, it was the most frequent in diabetic foot ulcers, confirming the low rate of nasal carriage in diabetics relative to other CCs (Dunyach-Remy et al., 2017).

A history of neurological disease has already been reported to be a risk factor for *S. aureus* CC398 blood-stream infections (Bouiller et al., 2016). This finding raises the question of hospital cross transmission to patients with a high level of care and poor autonomy. However, patients were hospitalized in different wards, and not specifically in the same period.

### 4.5. Limitations of the study

Our study had several limitations. First, epidemiological studies have defined three main categories of *S. aureus* carriers: intermittent carriers, persistent carriers, and non-carriers (Mehraj et al., 2016). Persistent carriers are characterized by certain host genetic factors and no clearance of *S. aureus* from the nose, with a high risk of infection (Verhoeven, 2013). Among 52 patients with *S. aureus* carriage and a repeat swab, 37 (71%) showed persistent positive *S. aureus* nasal carriage on day 7. Our study did not differentiate between intermittent and persistent carriers. For patients whose first sample was negative, it was difficult to interpret whether the second positive sample was due to strain acquisition or intermittent carriage. However, the rate of *S. aureus* CC398 nasal colonization was similar to that of blood-stream infection in our hospital.

Second, we did not use broth enhancement or PCR to detect *S. aureus*. Indeed, broth enhancement before plated on specific media could increase sensitivity of *S. aureus* detection. However, most of studies were performed for MRSA carriage, with few data on MSSA. It could be therefore possible that the prevalence of *S.aureus* nasal carriage had been under estimated (Safdar et al., 2003; Palavecino, 2020).

Third, the calculated number of patients to include in our study was 1,000 in each group. However, due to the COVID-19 pandemic, inclusion of patients was interrupted for 3 months. Nonetheless, the higher prevalence of *S. aureus* CC398 than expected did not modify the precision of the prevalence estimate.

Fourth, apart vegetarian status, we did not collected dietary regiment of patients included in the study. However, whereas transmission of *S. aureus* from livestock is associated with close contact to animal, the risk of transmission by food is very low, notably because meat is consumed cooked. Moreover, the European Food Safety Authority considered that the risk of infection of persons handling meat is very low (Bortolaia et al., 2016).

## 5. Conclusion

We show a high prevalence of *S. aureus* CC398 among hospitalized patients and a healthy community population, reaching approximately 20% in both cohorts. Factors associated with nasal carriage of *S. aureus CC398* were mainly general preconditions in both cohorts (age, smoking, history of neurological disease, absence of diabetes). Occupational expositional was not related. The high rate of erythromycin resistance may play a role in strain selection, particularly in patients who have taken antibiotics in the past year. Genotypic and phenotypic factors of this strain should be evaluated to better explain its spread in our region.

## Data availability statement

The data presented in the study are deposited in the NCBI/BioProject repository, accession number PRJNA901657.

## Ethics statement

The studies involving human participants were reviewed and approved by the protocol was approved by the Research Board of CPP Sud-Ouest et Outre-Mer 1 (CPP 1–18-92/SI 1780). The patients/participants provided their written informed consent to participate in this study.

## Author contributions

KB, HG-H, DH, CC, and XB: conceptualization. HG-H: methodology and formal analysis. KB and AZ: investigation and writing-original draft. XB and DH: resources. HG-H, DH, CC, and XB: writing–review and editing. XB and CC: supervision. KB: funding acquisition. All authors contributed to the article and approved the submitted version.

## Funding

This study was supported by the Region Bourgogne Franche-Comté (Appel à projet amorçage).

## Conflict of interest

The authors declare that the research was conducted in the absence of any commercial or financial relationships that could be construed as a potential conflict of interest.

## Publisher’s note

All claims expressed in this article are solely those of the authors and do not necessarily represent those of their affiliated organizations, or those of the publisher, the editors and the reviewers. Any product that may be evaluated in this article, or claim that may be made by its manufacturer, is not guaranteed or endorsed by the publisher.
