# Supplementary material for: Epidemiology and risk factors of nasal carriage of Staphylococcus aureus CC398 in two distinct cohorts in France
Source: Front Microbiol. 2022 Dec 20;13:1068420. doi: 10.3389/fmicb.2022.1068420 (PMC9807596; doi:10.3389/fmicb.2022.1068420)
Supplement: Supplementary file 1 [file Table_1.DOCX]

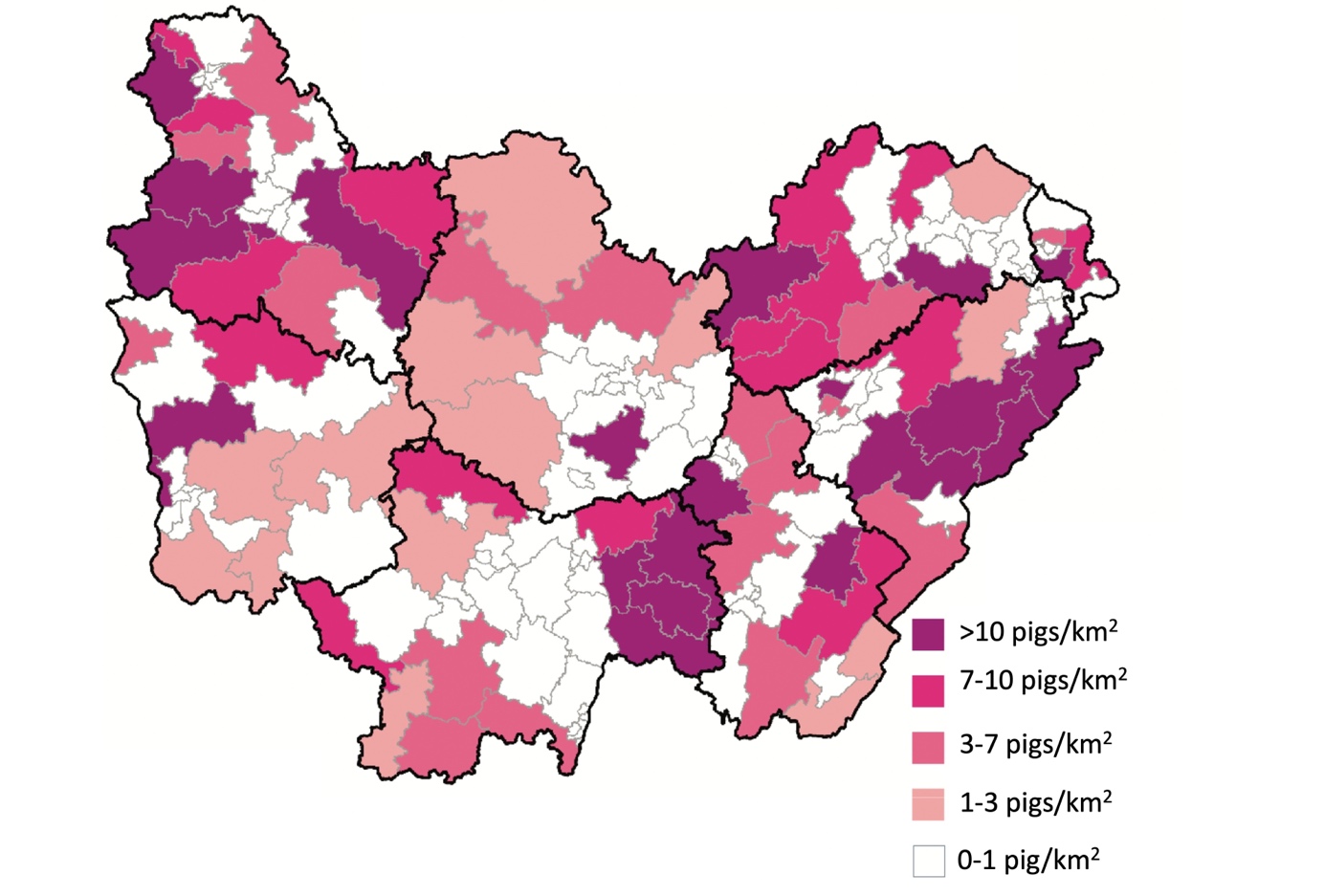


Site of nasal swab collection

Figure 1. Cities of nasal swab collection and pig density (pigs/km2) in Franche-Comté region. Figure adapted from <https://bourgognefranchecomte.chambres-agriculture.fr/fileadmin/user_upload/Bourgogne-Franche-Comte/061_Inst-Bourgogne-Franche-Comte/CRABFC/OPABFC_2018/10-Porcin.pdf> (2017)
